# Demographics and Outcomes of Spine Surgery in Octogenarians and Nonagenarians: A Comparison of the National Inpatient Sample, MarketScan and National Surgical Quality Improvement Program Databases

**DOI:** 10.7759/cureus.6195

**Published:** 2019-11-19

**Authors:** Siddharth Bhargava, Mayur Sharma, Nicholas Dietz, Joseph Dettori, Beatrice Ugiliweneza, Miriam Nuno, Maxwell Boakye, Doniel Drazin

**Affiliations:** 1 Neurosurgery, Albany College of Medicine, Albany, USA; 2 Neurosurgery, University of Louisville School of Medicine, Louisville, USA; 3 Evidence Practice Center, Spectrum Research, Tacoma, USA; 4 Statistics, University of California, Davis, USA; 5 Medicine, Pacific Northwest University of Health Sciences, Yakima, USA

**Keywords:** retrospective, database, spine surgery, octogenarians, nonagenarians

## Abstract

Introduction

Despite the increasing use of national databases to conduct spine research, questions remain regarding their study validity and consistency. This study tested for similarity and inter-database reliability in reported measures between three commonly used national databases.

Methods

International Classification of Diseases, 9th edition (ICD-9) codes were used to identify elderly (80-100 years) who underwent spine surgery patients in Truven Health Analytics MarketScan® claims database, National (Nationwide) Inpatient Sample (NIS) discharge database and National Surgical Quality Improvement Program (NSQIP) database (2006-2016). Patient baseline characteristics, comorbid status, insurance enrollment, and outcomes were queried and compared.

Results

We analyzed 15,105 MarketScan, 40,854 NIS, and 7682 NSQIP patients between ages 80 to 100 years (median, 82 years) who underwent spine surgeries during the study period. A majority of patients in both MarketScan and NIS were insured by Medicare (97% vs. 94%). Patients in MarketScan had lower comorbidity scores (comorbidity, 0-2) compared to those in NIS and NSQIP databases. The most common diagnosis was spinal stenosis in MarketScan (54.4%), NIS (54.6%), and NSQIP databases (65.2%). Fusion was the most common procedure performed in MarketScan (48.9%) and NIS databases (46.2%), whereas decompression (laminectomy/laminotomy) was the most common procedure in the NSQIP database (51.84%). In-hospital complications (any) were 6.5% in the MarketScan cohort, 5.3% in the NIS, and 2.02% in the NSQIP cohort. In terms of 30-day complications (any), the MarketScan database reported higher complications rate (12.7%) compared to the NSQIP database (5.08%). In-hospital mortality was slightly higher in the NIS database (0.32%) compared to MarketScan (0.21%) and NSQIP database (0.2%). MarketScan and NIS databases showed an increased risk of complications with increasing age, whereas NIS and NSQIP showed increasing complications with a higher number of comorbidities. Male gender had higher complication at 30-day post-discharge using MarketScan and NSQIP database.

Conclusions

Patients in the NSQIP and NIS database have more comorbidities; patients in the MarketScan database had the highest number of perioperative and 30-day post-discharge complications with the highest number of fusion procedures performed. Patients in the NSQIP database had the lowest number of fusion procedures and complication rates. As databases gain popularity in spine surgery, clinicians and reviewers should be cautious in generalizing results to whole populations and pay close attention to the population being represented by the data from which the statistical significance was derived.

## Introduction

Researchers must cross a multitude of barriers to document a sufficiently large cohort to study rare diseases and procedures [1]. National databases allow expedited investigation of widespread trends and demographics for clinical interpretation [1-4]. Retrospective analysis of focused cohorts provides clinicians with opportunities to understand their patient population comprehensively and implement care delivery strategies to improve outcomes. This is a crucial step in preventing medical errors and improving the quality of care [1,5]. As database studies gain traction among researchers; it is essential to ensure external validity by understanding key characteristics and composition of the databases, which is made difficult due to limited granularity of clinical circumstances, before confidently generalizing the results to the clinical population [6].

One such focused population that remains difficult to be studied prospectively is the elderly who undergo spine procedures. Wary surgeons refrain from operating on this population due to the prevalence of comorbidities and frailty [7,8]. However, certain advances, including minimally invasive surgery and enhanced recovery after surgery (ERAS), have drastically improved the procedural outcomes. A possible solution is to retrospectively extract outcomes from databases to assess the viability of spine surgery in the elderly population. The present study reports the differences in three commonly used databases, MarketScan, National Inpatient Sample (NIS), and National Surgical Quality Improvement Program (NSQIP) in regards to patient demographics, complications, and outcomes following spine surgery in octogenarians and nonagenarians. 

## Materials and methods

Data sources

The Truven Health Analytics MarketScan® claims database collects participant information from Commercial Claims and Encounters, Medicare Supplemental and Coordination of Benefits and Medicaid databases. Insurance enrollment, inpatient and outpatient utility, and claims and costs are provided and organized based on 150 payers in the US from employer-based plans [9]. A neurology/neurosurgery custom-dataset obtained from MarketScan spanning from 2000-2012 was used. Medicare in MarketScan is Medicare Supplemental (also called Medigap). These patients are those on Medicare who can afford to take supplemental insurance to cover some things that Medicare doesn't cover. 

The Healthcare Costs and Utilization Project (HCUP) NIS is the largest all-payer inpatient database that collates discharge patient information on all inpatient admissions in non-federal US hospitals. A stratified random sampling technique of the hospitals and patients produces a representative 20% subsample, which can be generalized to the American medical community [10]. The Elixhauser comorbidity data was implemented to NIS in 1998, which allows for calculating risk adjustments through the database [11]. We extracted a custom dataset spanning from 2000 to 2012 [10]. The NIS data for this study was adapted from Drazin et al. with permission [12]. 

National Surgical Quality Improvement Program (NSQIP) is a well-recognized nationally validated outcome-based database introduced by the American College of Surgeons (ACS) to improve the quality of surgical care. Data is extracted using the International Classification of Diseases (ICD) 9/10 and Current Procedural Terminology (CPT) codes using this database and include comorbidities and postoperative outcomes. 

Data extraction

The study population was composed of a retrospective cohort study of patients undergoing spine surgery procedures for spinal stenosis in the Truven Health Analytics MarketScan® database, NIS database, and NSQIP database from 2006-2016. Patient extraction was performed using the International Classification of Diseases, 9th edition (ICD-9) coding system (for all databases), and the Current Procedural Terminology, 4th edition (CPT-4) (for MarketScan only). MarketScan is a longitudinal database. For this study, the first occurring hospitalization, satisfying the extraction conditions, was used for patient characteristics and most outcomes. Patient baseline characteristics included: age, gender, comorbid status, insurance type, and primary procedure. Outcome measures included: in-hospital complication and mortality risks, length of stay (LOS), and stratified in-hospital complication risks. Multivariable analysis assessed the association of baseline and patient characteristics with perioperative complications.

Data was queried to identify patients between the ages of 80-100 years who underwent spinal decompression (ICD-9 codes: 03.0, 03.09), discectomy (ICD-9 codes: 80.50, 80.51), or spinal fusion (ICD-9 codes: 81.0, 81.00, 81.01, 81.02, 81.03, 81.04, 81.05, 81.06, 81.07, 81.08). Primary diagnoses included spinal stenosis (ICD-9 codes: 723.0, 724.0, 724.00, 724.01, 724.02, 724.09), claudication (ICD-9 codes: 724.03), disc herniation (ICD-9 codes: 722.0, 722.10, 722.11), and disc protrusion (ICD-9 codes: 722.30, 722.31, 722.32, 722.51, 722.52, 722.71, 722.72, 722.73). Supplemental tables summarize ICD-9/10 and CPT codes used to extract data from these databases (Appendices). Patients younger than age 80 years, older than 100, and those undergoing vertebroplasty and kyphoplasty (augmentation procedures) were excluded.

Statistical analysis

Patient characteristics were summarized using means and standard deviation (for continuous variables) and counts and percentage (for categorical variables). Differences were considered significant if p<0.0001. Each outcome (mortality, complications, and length of stay), within each database, was analyzed in a multivariable analysis including four variables (age at diagnosis, gender, comorbid state, and procedural type). Results were presented in terms of odds ratio (OR) or relative risk (RR) with associated 95% confidence interval.

## Results

A total of 63,641 octogenarians and nonagenarians who underwent spinal decompression, discectomy, or fusion surgery for spinal stenosis were identified from all the databases. The baseline patient characteristics and procedure outcomes were compared between the 15,105 MarketScan, 40,854 NIS, and 7682 NSQIP patients. Calculated odds-ratio of experiencing a perioperative complication during index hospitalization is presented in Table 1.

**Table 1:** Adjusted Odds Ratio (OR) of Complications at Index Hospitalization and 30 Days After Admission

		Index Hospital Complication			30-day from admission Complication	
		MarketScan	National Inpatient Sample	National Surgical Quality Improvement Program	MarketScan	National Surgical Quality Improvement Program
Variable						
Age, 1 year increase		1.05 (1.03, 1.08)	1.05 (1.04, 1.07)	1.01 (0.94, 1.09)	1.04 (1.02, 1.06)	1.02 (0.98, 1.07)
Gender (ref: male)						
	Female	0.83 (0.73, 0.95)	0.73 (0.67, 0.8)	0.78 (0.53, 1.13)	0.81 (0.73, 0.89)	0.75 (0.61, 0.93)
Comorbidities group (ref: 0)						
	1	0.91 (0.78, 1.06)	1.07 (0.94, 1.22)	1.15 (0.49, 2.71)	0.98 (0.88, 1.1)	1.06 (0.67, 1.67)
	2	1.15 (0.96, 1.37)	1.54 (1.35, 1.76)	2.16 (0.96, 4.87)	1.21 (1.06, 1.39)	1.63 (1.05, 2.52)
	3-8	1.29 (0.96, 1.74)	2.52 (2.16, 2.94)	2.45 (1.07, 5.61)	1.47 (1.18, 1.83)	2.69 (1.74, 4.17)
Diagnosis (ref: Spinal stenosis)						
	Disc herniation	0.85 (0.68, 1.07)	0.88 (0.76, 1.03)	0.57 (0.26, 1.22)	0.9 (0.76, 1.06)	0.82 (0.57, 1.18)
	Disc protrusion	1.2 (1.01, 1.43)	1.2 (1.07, 1.35)	1.4 (0.84, 2.34)	1.16 (1.02, 1.33)	0.87 (0.61, 1.23)
	Degeneration	0.9 (0.74, 1.11)	0.86 (0.75, 0.99)	0.87 (0.49, 1.56)	1.01 (0.87, 1.17)	1.06 (0.78, 1.45)
Procedure (ref: fusion)						
	Decompression	0.58 (0.5, 0.68)	0.6 (0.55, 0.67)	0.48 (0.32, 0.72)	0.64 (0.57, 0.71)	0.55 (0.43, 0.69)
	Discectomy	0.61 (0.47, 0.81)	0.69 (0.59, 0.8)	0.45 (0.25, 0.82)	0.71 (0.59, 0.87)	0.54 (0.39, 0.74)

Demographics and patient characteristics

The median age was 82 years (IQR: 81-85) in all the databases. MarketScan and NIS databases found females to have undergone frequent spine surgeries compared to males (53% vs. 54%, respectively), whereas the NSQIP database showed an equal proportion of males and females undergoing surgeries, Table 2. A majority of patients in both MarketScan and NIS were insured by Medicare (97% vs. 94%). Medicaid was more commonly reported with MarketScan enrollees compared to NIS enrollees (3.1% vs. 0.27%). Because NIS accumulates data on all payers, it has the ability to report commercial/private (4.7%) and other methods of payments (1.08%). Patients in MarketScan had lower comorbidity scores (comorbidity: 0-2) compared to those in NIS and NSQIP databases. Patients with 3+ comorbidities constituted 4% in MarketScan, 9% in NIS and 22% in NSQIP databases. Hypertension was the most common comorbidity, with a median of one comorbidity across the MarketScan and NIS databases (NSQIP: median, 2 comorbidities).

**Table 2:** Patient Characteristics (2006-2016)

		MarketScan	National Inpatient Sample	National Surgical Quality Improvement Program
Variable		N=15105	N=40854	N=7682
Age				
	Mean (SD)	83.1 (2.8)	83.1 (2.8)	82.9 (2.5)
	Median (IQR)	82 (81, 85)	82 (81, 85)	82 (81, 85)
	Range (min-max)	80-103	80-110	80-89
Gender, n (%)				
	Female	7974 (52.79%)	22224 (54.43%)	3835 (49.93%)
Race, n (%)				
	White		31396 (76.85%)	6600 (85.92%)
	Black		1022 (2.5%)	236 (3.07%)
	Other/unknown		8436 (20.65%)	846 (11.01%)
Type of Insurance, n (%)				
	Commercial/private		1933 (4.73%)	
	Medicaid	468 (3.1%)	109 (0.27%)	
	Medicare	14637 (96.9%)	38370 (93.92%)	
	Other		442 (1.08%)	
Comorbidities group, n (%)				
	0	5551 (36.75%)	7842 (19.2%)	788 (10.26%)
	1	6141 (40.66%)	18479 (45.23%)	2474 (32.21%)
	2	2764 (18.3%)	10838 (26.53%)	2668 (34.73%)
	3-10	649 (4.3%)	3695 (9.04%)	1752 (22.81%)
Comorbidities details, n (%)				
1	Anemia	2080 (13.77%)	8018 (19.63%)	2683 (34.93%)
2	Bleeding disorder	222 (1.47%)	927 (2.27%)	283 (3.68%)
3	COPD	955 (6.32%)	3402 (8.33%)	448 (5.83%)
4	Diabetes	2480 (16.42%)	9169 (22.44%)	1564 (20.36%)
5	Hypertension	7356 (48.7%)	26824 (65.66%)	6026 (78.44%)
6	Morbid obesity	118 (0.78%)	454 (1.11%)	125 (1.63%)
7	Obesity	301 (1.99%)	2057 (5.04%)	2114 (27.52%)
8	Smoking	179 (1.19%)	1059 (2.59%)	280 (3.64%)
	Any one of above	9554 (63.25%)	33012 (80.8%)	6894 (89.74%)
Sum of above				
	Mean (SD)	0.9 (0.9)	1.3 (0.9)	1.8 (1)
	Median (IQR)	1 (0, 1)	1 (1, 2)	2 (1, 2)
	Range (min-max)	0-5	0-6	0-6

The primary diagnoses and procedures performed across the databases are presented in Tables 3, 4. The most common diagnosis was spinal stenosis in MarketScan (54.4%), NIS (54.6%), and NSQIP databases (65.2%). Fusion was the most common procedure performed in MarketScan (48.9%) and NIS databases (46.2%), whereas decompression (laminectomy/laminotomy) was the most common procedure in NSQIP database (51.84%). Discectomy for spinal stenosis was the least common procedure performed across the databases. 

**Table 3:** Primary Diagnoses and Procedures of Cohorts

		MarketScan	National Inpatient Sample	National Surgical Quality Improvement Program
Variable		N=15105	N=40854	N=7682
Diagnosis, n (%)				
	Spinal stenosis	8216 (54.39%)	22328 (54.65%)	5011 (65.23%)
	Disc herniation	2474 (16.38%)	6967 (17.05%)	970 (12.63%)
	Disc protrusion	2355 (15.59%)	6207 (15.19%)	763 (9.93%)
	Degeneration	2060 (13.64%)	5352 (13.1%)	938 (12.21%)
Procedures, n (%)				
	Fusion	7386 (48.9%)	18863 (46.17%)	2185 (28.44%)
	Decompression (Laminectomy/laminotomy)	6067 (40.17%)	15670 (38.36%)	3982 (51.84%)
	Discectomy	1652 (10.94%)	6321 (15.47%)	1515 (19.72%)

**Table 4:** Diagnosis, Procedures, and Complications

		MarketScan				National Inpatient Sample				National Surgical Quality Improvement Program			
		Fusion	Decompression	Discectomy	p-value	Fusion	Decompression	Discectomy	p-value	Fusion	Decompression	Discectomy	p-value
		n=7386	n=6067	n=1652		n=18863	n=15670	n=6321					
Diagnosis, n (%)													
	Spinal stenosis	3171 (42.93%)	4742 (78.16%)	303 (18.34%)		8249 (43.73%)	12880 (82.2%)	1199 (18.97%)		1136(51.99%)	3120 (78.35%)	755 (49.83%)	
	Disc herniation	760 (10.29%)	527 (8.69%)	1187 (71.85%)		1929 (10.23%)	634 (4.05%)	4404(69.67%)		163 (7.46%)	285 (7.16%)	522 (34.46%)	
	Disc protrusion	1672 (22.64%)	548 (9.03%)	135 (8.17%)		4195 (22.24%)	1458 (9.3%)	554 (8.76%)		390(17.85%)	270 (6.78%)	103 (6.8%)	
	Degeneration	1783 (24.14%)	250 (4.12%)	27 (1.63%)		4490 (23.8%)	698 (4.45%)	164 (2.59%)		496 (22.7%)	307 (7.71%)	135 (8.91%)	
In Hospital Complications, n (%)													
1	Acute Kidney Injury	207 (2.8%)	108 (1.78%)	36 (2.18%)	0.0004	685 (3.63%)	332 (2.12%)	150 (2.37%)	<0.0001	4 (0.18%)	4 (0.1%)	2 (0.13%)	0.6903
2	Any surgical site infection	29 (0.39%)	13 (0.21%)	4 (0.24%)	0.155	35 (0.19%)	26 (0.17%)	12 (0.19%)	0.8882	8 (0.37%)	2 (0.05%)	3 (0.2%)	0.0148
3	Cardiac Arrest	17 (0.23%)	14 (0.23%)	2 (0.12%)	0.6679	48 (0.25%)	26 (0.17%)	5 (0.08%)	0.014	6 (0.27%)	2 (0.05%)	1 (0.07%)	0.0389
4	Deep vein thrombosis	64 (0.87%)	39 (0.64%)	12 (0.73%)	0.327	127 (0.67%)	77 (0.49%)	33 (0.52%)	0.069	12 (0.55%)	17 (0.43%)	4 (0.26%)	0.4269
5	Myocardial Infarction	64 (0.87%)	38 (0.63%)	11 (0.67%)	0.252	164 (0.87%)	86 (0.55%)	31 (0.49%)	0.0002	15 (0.69%)	13 (0.33%)	2 (0.13%)	0.0188
6	Pneumonia	170 (2.3%)	72 (1.19%)	14 (0.85%)	<0.0001	307 (1.63%)	161 (1.03%)	58 (0.92%)	<0.0001	32 (1.46%)	16 (0.4%)	4 (0.26%)	
7	Pulmonary Embolism	46 (0.62%)	14 (0.23%)	5 (0.3%)	0.0018	68 (0.36%)	42 (0.27%)	8 (0.13%)	0.0092	8 (0.37%)	9 (0.23%)	1 (0.07%)	0.1762
8	Stroke	67 (0.91%)	48 (0.79%)	6 (0.36%)	0.0805	89 (0.47%)	48 (0.31%)	21 (0.33%)	0.0357	6 (0.27%)	3 (0.08%)	2 (0.13%)	0.1399
9	Wound Dehiscence	9 (0.12%)	3 (0.05%)	0 (0%)	0.1593	17 (0.09%)	13 (0.08%)	2 (0.03%)	0.3432	1 (0.05%)	2 (0.05%)		0.6892
	Any one of the above	602 (8.15%)	309 (5.09%)	78 (4.72%)	<0.0001	1295 (6.87%)	684 (4.37%)	280 (4.43%)	<0.0001	76 (3.48%)	61 (1.53%)	18 (1.19%)	
30-day Complications, n (%)													
1	Acute Kidney Injury	311 (4.21%)	160 (2.64%)	53 (3.21%)	<0.0001					6 (0.27%)	14 (0.35%)	7 (0.46%)	0.6384
2	Any surgical site infection	201 (2.72%)	119 (1.96%)	25 (1.51%)	0.0011					48 (2.2%)	54 (1.36%)	19 (1.25%)	0.0214
3	Cardiac Arrest	30 (0.41%)	15 (0.25%)	5 (0.3%)	0.2731					7 (0.32%)	7 (0.18%)	3 (0.2%)	0.5011
4	Deep vein thrombosis	181 (2.45%)	104 (1.71%)	32 (1.94%)	0.011					29 (1.33%)	41 (1.03%)	12 (0.79%)	0.2811
5	Myocardial Infarction	98 (1.33%)	61 (1.01%)	16 (0.97%)	0.1662					20 (0.92%)	21 (0.53%)	7 (0.46%)	0.1209
6	Pneumonia	328 (4.44%)	161 (2.65%)	44 (2.66%)	<0.0001					49 (2.24%)	36 (0.9%)	8 (0.53%)	
7	Pulmonary Embolism	105 (1.42%)	44 (0.73%)	19 (1.15%)	0.0006					15 (0.69%)	19 (0.48%)	6 (0.4%)	0.4148
8	Stroke	119 (1.61%)	88 (1.45%)	16 (0.97%)	0.1437					10 (0.46%)	8 (0.2%)	4 (0.26%)	0.193
9	Wound Dehiscence	54 (0.73%)	33 (0.54%)	6 (0.36%)	0.1466					5 (0.23%)	3 (0.08%)	3 (0.2%)	0.2565
	Any one of the above	1125 (15.23%)	630 (10.38%)	174 (10.53%)	<0.0001					159 (7.28%)	172 (4.32%)	59 (3.89%)	

Length of hospital stay, complications, and mortality 

The median length of hospital stay was similar across the cohorts (3 days) with IQR of 2-4 days in MarketScan, 2-5 days in NIS, and 1-4 days in NSQIP database. In-hospital complications (any) were 6.5% in the MarketScan cohort, 5.5% in the NIS cohort, and 2% in the NSQIP cohort, with the most common being acute renal injury followed by pneumonia in both MarketScan and NIS database. Whereas pneumonia followed by deep vein thrombosis (DVT) were common complications in NSQIP database. In terms of 30-day complications (any), MarketScan database reported higher complications rate (12.7%) compared to NSQIP database (5.08%) and pneumonia (3.53%) was the most common complication in MarketScan database, whereas surgical site infection (1.58%) was the most common in NSQIP database. In-hospital mortality was slightly higher in the NIS database (0.32%) compared to MarketScan (0.21%) and NSQIP database (0.2%), Table 5.

**Table 5:** Length of Stay, In-hospital Mortality and Complications

		MarketScan	National Inpatient Sample	National Surgical Quality Improvement Program
Variable		N=15105	N=40854	N=7682
Length of stay, days				
	Mean (SD)	3.6 (3.5)	3.8 (3.5)	3.5 (3.9)
	Median (IQR)	3 (2, 4)	3 (2, 5)	3 (1, 4)
	Range (min-max)	1-102	0-84	0-67
In-hospital mortality, n (%)				
	Mortality, yes	32 (0.21%)	132 (0.32%)	15 (0.2%)
In Hospital Complications, n (%)				
1	Acute Kidney Injury	351 (2.32%)	1167 (2.86%)	10 (0.13%)
2	Any surgical site infection	46 (0.3%)	73 (0.18%)	13 (0.17%)
3	Cardiac Arrest	33 (0.22%)	79 (0.19%)	9 (0.12%)
4	Deep vein thrombosis	115 (0.76%)	237 (0.58%)	33 (0.43%)
5	Myocardial Infarction	113 (0.75%)	281 (0.69%)	30 (0.39%)
6	Pneumonia	256 (1.69%)	526 (1.29%)	52 (0.68%)
7	Pulmonary Embolism	65 (0.43%)	118 (0.29%)	18 (0.23%)
8	Stroke	121 (0.8%)	158 (0.39%)	11 (0.14%)
9	Wound Dehiscence	12 (0.08%)	32 (0.08%)	3 (0.04%)
	Any one of the above	989 (6.55%)	2259 (5.53%)	155 (2.02%)
30-day Complications, n (%)				
1	Acute Kidney Injury	524 (3.47%)		27 (0.35%)
2	Any surgical site infection	345 (2.28%)		121 (1.58%)
3	Cardiac Arrest	50 (0.33%)		17 (0.22%)
4	Deep vein thrombosis	317 (2.1%)		82 (1.07%)
5	Myocardial Infarction	175 (1.16%)		48 (0.62%)
6	Pneumonia	533 (3.53%)		93 (1.21%)
7	Pulmonary Embolism	168 (1.11%)		40 (0.52%)
8	Stroke	223 (1.48%)		22 (0.29%)
9	Wound Dehiscence	93 (0.62%)		11 (0.14%)
	Any one of the above	1929 (12.77%)		390 (5.08%)

Both MarketScan and NIS databases showed an increased risk of complication with increasing age, whereas NIS and NSQIP databases showed increased complications with an increasing number of comorbidities. Male gender had higher complication during index hospitalization using MarketScan and NIS database, and 30-day post-discharge using MarketScan and NSQIP database. Using the MarketScan database, patients with 2 and 3+ comorbidities had 1.21 (1.06, 1.39), and 1.47 (1.18, 1.83) higher odds of experiencing a complication compared to those with no comorbidities, respectively at 30 days after hospitalization. In terms of diagnosis, patients with disc protrusion had a higher risk of complications during index hospitalization and 30 days post-discharge compared to those with a diagnosis of spinal stenosis. Compared to fusion, patients undergoing decompression and discectomy had lower odds of developing complications during index hospitalization [MarketScan database: Decompression: 0.58 (0.5-0.68); Discectomy: 0.61 (0.47-0.81)] and [NIS database: Decompression: 0.6 (0.55-0.67); Discectomy: 0.69 (0.59- 0.8) discectomy] and 30 days post-discharge, Table 5. 

## Discussion

Incorporation of national databases into research has substantially increased in the past few years [2,3,6,13,14]. Although these large sample sizes offer researchers opportunities to investigate rare diseases, the statistically significant results are nevertheless susceptible to type I errors, or false-positive results [1]. Therefore, it is essential to understand the observational and retrospective nature of the database and its sample populations prior to generalizing its outcomes to the total population when given statistically significant results. To our knowledge, this study is the first to compare outcomes and demographics of elderly patients undergoing spine surgery in three commonly used databases.

Demographics and outcomes

In comparing MarketScan, NIS, and NSQIP databases, our study found several differences between the cohorts. Compared to the NIS and NSQIP cohort, the MarketScan cohort was healthier, possibly owing to a larger group of participants with fewer documented comorbidities. Although nearly half the patients’ primary procedure was decompression in all three databases, a slightly larger proportion of the MarketScan cohort underwent fusion compared to the NIS and NSQIP cohort. The outcomes of the database reflect the cohort composition. The mortality rates between the sampled populations were not significantly different, which could be due to the overall low mortality rates of spine surgery. In addition, higher rates of complications have been associated with patients undergoing fusion surgery, especially in the elderly, and with those affected by a higher number of comorbidities [15-20]. This could explain why fusion surgery was performed more frequently in the healthier MarketScan population, compared to the sicker NIS population with a higher number of comorbidities. 

Differences in the national administrative databases

Non-uniform methodology of these databases can uncover difficulties in generalizing results and thus drawing clinical significance. Crucial differences can arise from each database’s sampling methods. Truven Health Analytics MarketScan® database compiles its samples from claims of employees, Medicare-eligible retirees, early retired, Consolidated Omnibus Budget Reconciliation Act (COBRA) participants and their dependents enrolled through large US corporations in the private sector [9,21]. In contrast, HCUP NIS collects a stratified systematic sample from all HCUP hospitals, which is equivalent to 20% of all discharges from community hospitals in the United States [10,14]. Based on the method used to collect the cohort sample, NIS is most likely representative of national means and the US population. However, NIS contains information related to hospital discharges only. MarketScan readily offers outpatient visit information, allowing for better understanding in longitudinal aspects for investigation. Since MarketScan collates participants from those insured by large US corporations, their sample may be limited to specific geographic or socioeconomic groups [21]. It can be argued that because MarketScan databases cover participants who were insured through large US corporations, they may not be as representative or comparable of the general US population. Whereas, NSQIP is a nationally validated program forwarded by the American College of Surgeons (ACS) aimed to improve the quality of surgical care by providing tools to participating hospitals. 

Overall, while it is not surprising to report that advanced aged participants are predominantly enrolled in Medicare, discerning discrepant trends allows patients to choose clinically and economically sound providers to anticipate healthcare costs. An arsenal of comprehensive variables is necessary to streamline patients’ experiences and outcomes [22]. Due to its limited collation of participant data from only US corporations, MarketScan is theoretically unable to present a cohort that is characteristic of the whole US population. Nonetheless, studies examining the quality of NIS data found discrepancies when comparing results derived from patient charts and administrative data from ICD-9 billing codes [1,2,23]. Furthermore, billing-codes are variable on the interpretation and accuracy of the operator (trained vs. naïve) as well as external political and economic pressures leading to variability in application of different codes for a similar procedure in different databases [1]. 

Since these databases have numerous overlapping variables, and no single database contains all variables, multiple database approach may help compensate for their respective weaknesses. Buckland et al. showed that national databases such as NIS and NSQIP did not capture a similar patient population when compared to physician managed database (PMD) in patients underusing surgery for adult spinal deformity [24]. This difference can be attributed to the referral pattern and selection bias in the PMD cohort. Similarly, Bohl et al. showed that NIS and NSQIP databases gave different results (complications and comorbidities) in patients with hip fractures [25]. In concordance to these studies, we found that 30-day post-discharge complications varied significantly between MarketScan (12.77%) and NSQIP database (5.1%). 

According to comorbidity scores alone, NIS and NSQIP patients were less healthy than their MarketScan counterparts. In our study, we used Elixhauser comorbidity index for analysis in all three databases. Nonetheless, it is integral to question the comorbidity indices implemented for the analysis, as not all comorbidities are weighted equally among each index. The algorithm of Elixhauser comorbidity index was developed to predict the inpatient outcomes in hospitalized patients based on their acute and chronic conditions [11,26]. It has been demonstrated to predict the in-hospital mortality with respect to disease burden, especially after 30-days of hospitalization [27]. In contrast, the Charlson comorbidity index was designed to predict one-year mortality based on a patient’s comorbidities [28]. While both calculations are commonly utilized to discriminate for future mortality outcomes, Menendez et al. reported that the Elixhauser comorbidity method outperformed Charlson Index in regards to predicting inpatient outcomes after specifically orthopedic surgery [29]. Thus, inclusion and exclusion criteria for pertinent variables of candidate databases should be deliberated to identify the optimal database fitting study aims.

Differences yet similarity among databases 

It is important, however, to note that despite vastly different sample sizes, demographics, and collection methods, the primary and secondary results from the databases are not different. The large cohort sizes provide a means to obtain statistical significance that highlight minor differences, but these differences may not be clinically relevant. Additionally, not infrequently, clinicians afford too much attention to p-values, forgetting to vet the generalizability. Although minor differences are highlighted due to the power of the study, broadly, the results of these databases are moderately consistent with one another, suggesting precise results despite differing acquisition methods. Nonetheless, we caution clinicians from generalizing results of database studies. Although they theoretically should represent the population of the country through their sampling methods, generalizing this data to the total population may not be accurate due to the retrospective and observational nature of database studies, especially considering changing practices and advancing minimally invasive technologies. While the owners of the database may promise internal validity, we must be wary of assigning external validity to the total patient population.

Limitations and strengths 

This study has several limitations. First, the accuracy of our results depends largely on the accuracy and consistency of the reported diagnosis and procedure codes. Secondly, the inability to match patients between these three databases limits our capability to reason several of the discovered outcome-discrepancies. Specific patient profiles would allow analysis regarding adherence to evidence-based medicine and hospital guidelines, especially in standards with the geographical location [30]. One such finding includes the differences in stratified post-operative complications between the three databases. Although the most common specific complications were alike in the three databases, it is difficult to ascertain the discrepancies without additional granular clinical details. 

Notably, MarketScan and NSQIP can track patient data after the perioperative period. In contrast, NIS was limited to information accumulated during the immediate inpatient stay, thereby disallowing longitudinal comparison to determine superiority in that regard. Moreover, because both NIS and MarketScan were not designed to collect spine- or orthopedic-specific data, this study was limited to available variables. Reported improvements in the quality of life and activities of daily living following procedures would provide integral insight into necessary changes required to expand care delivery outcomes. As all databases offer different groups of patient characteristics and widely differ in their sample collection, we remain cognizant of the limitation in the generalizability of the comparison of results and databases.

## Conclusions

Even though the results of the three commonly used databases were not completely different, suggesting some consistency despite differing sampling methods, this study captures the discrepancies in the demographics of spine surgery. The disparities drive the variations observed in preoperative comorbid status and inpatient and long-term adverse events. Overall, it appears that the patients in the NSQIP and NIS database have more comorbidities, patients in the MarketScan database had the highest number of perioperative and 30-day post-discharge complications with the highest number of fusion procedures performed. Patients in the NSQIP database had the lowest number of fusion procedures and complication rates. Thus, researchers should be wary of generalizing results from sample populations onto total populations with retrospective, observational database study designs. Future studies may additionally benefit from different database approaches to supplement any vulnerabilities of the primary database.

## Appendices

**Table 6:** Summary of ICD-9/10 Primary Complication and Comorbidity Codes Utilized to Query Data from MarketScan, National Inpatient Sample and National Surgical Quality Improvement Program Databases International Classification of Diseases character 1 (i.e. E, J, N) refers to medical or surgical category designation and character 2 refers to body system.

Comorbidities	International Classification of Diseases-9 Code	International Classification of Diseases-10 Code
Congestive Heart Failure	39891','40201','40211','40291','40401','40403','40411','40413','40491', '40493','4254','4255','4257','4258','4259','428'	I099','I110','I130','I132','I255','I420','I425','I426','I427','I428', 'I429','I43','I50','P290'
Hypertension	401', '402','403','404','405'	I10', 'I11','I12','I13','I15'
Chronic Pulmonary Disease	4168','4169','490','491','492','493','494','495','496','500','501','502', '503','504','505','5064','5081','5088'	I278','I279','J40','J41','J42','J43','J44','J45','J46','J47','J60','J61', 'J62','J63','J64','J65','J66','J67','J684','J701','J703'
Diabetes	2500','2501','2502','2503', '2504','2505','2506','2507','2508','2509'	E100','E101','E109','E110','E111','E119','E120','E121','E129','E130', 'E131','E139','E140','E141','E149', 'E102','E103','E104','E105','E106','E107','E108','E112','E113','E114','E115', 'E116','E117','E118','E122','E123','E124','E125','E126','E127','E128','E132', 'E133','E134','E135','E136','E137','E138','E142','E143','E144','E145','E146', 'E147','E148'
Renal Failure	40301','40311','40391','40402','40403','40412','40413','40492','40493', '585','586','5880','V420','V451','V56'	I120','I131','N18','N19','N250','Z490','Z491','Z492','Z940','Z992'
Metastatic Cancer	196','197','198','199'	C77','C78','C79','C80'
Coagulopathy	286','2871','2873','2874','2875'	D65','D66','D67','D68','D691','D693','D694','D695','D696'
Obesity	2780'	E66'
Weight Loss	260','261','262','263','7832','7994'	E40','E41','E42','E43','E44','E45','E46','R634','R64'
Fluid and Electrolyte Disorders	2536','276'	E222','E86','E87'
Complications		
Renal	584.xx; 997.5	N17.x; N99.89
Cardiac	410.xx; 997.1	I21.x; I97.7xx; I97.8xx
Nervous system Complication	997.09	G978.x
Stroke/CVA with neurological deficit	434.01; 434.11; 434.91	I63.3x; I63.4x; I63.5x;
DVT and pulmonary embolism	415.xx; 451.xx; 452; 453.xx	I26.xx; I80.xx; I81.xx; I82.xx
Pulmonary	518.4; 518.5; 518.8x; 997.3x	J81.0; J80; J95.1; J95.2; J95.3; J95.8xx; J96.xx
Infection	595.0; 595.9; 599.0	N30.00; N30.01; N30.90; N30.91; N39.0
Wound	998.32; 998.51; 998.6; 998.81; 998.83	T81.31xx; T81.4xx; T81.8xxx
Pneumonia	481.xx; 482.xx; 486.xx	J13-J18.x

**Table 7:** Summary of ICD-9/10 Primary Procedure Codes Utilized to Query Data from MarketScan, National Inpatient Sample and National Surgical Quality Improvement Program Databases International Classification of Diseases character 1 (i.e. E, J, N) refers to medical or surgical category designation and character 2 refers to body system, character 3 (i.e. 0B, 0G) refers to root operation.

Procedures	International Classification of Diseases-9 Code	International Classification of Diseases-10 Code	Current Procedural Terminology Code	Description for International Classification of Diseases-9
Decompression				
	03.09 (this includes all levels)	0RBxyZZ(X=0,1,4,6,A); 0SBxyZZ(X=0,3); -Excision	22102, 22114, 22207,22214, 22224	Other exploration and decompression of spinal canal
		00NxyZZ(x=W, X,Y), -Release	63005, 63012, 63017,63030, 63035,	
		009xy0Z, 009xyZZ(x=T, W, X,Y); 009U00Z, 009U0ZZ-Drainage		
		00JV0ZZ, 00JU0ZZ -Inspection	63042, 63047, 63056,63087, 63090,	
	80.50 -Excision or destruction of intervertebral disc, unspecified		63102, 0171T, 0202T, 0221T, 63200,	Excision or destruction of intervertebral disc, unspecified
			63252, 63267, 63272,63277, 63282,	
	80.51 -Excision of intervertebral disc	0RBxyZZ(X=3,5,9,B); 0SBxyZZ(X=2,4); -Excision	63387, 63302,63303, 63306, 63307,	Excision of intervertebral disc
		0RTx0ZZ(X=3,4,5,9,B); 0STx0ZZ(X=2,4); -Resection		
	80.59 -Other destruction of intervertebral disc	0R5x0ZZ(X=3,5,9,B); 0S5xyZZ(X=2,4); -Destruction (+ icd10 for 80.51)		Other destruction of intervertebral disc
	84.6x -disc replacement	0RRx0JZ(x=3,5,9,B); 0SRx0JZ(x=2,4); 0RWxyJZ(x=3,5,9,B); 0SWxyJZ(x=2,4);		
		0RPx0JZ+0RRx0JZ(x=3,5,9,B); 0SPx0JZ+0SRx0JZ(x=2,4)		
Exclude	81.65	0PU33JZ, 0PU34JZ, 0PU43JZ, 0PU44JZ, 0QU03JZ, 0QU04JZ, 0QU13JZ, 0QU14JZ		Percutaneous vertebroplasty
Exclude	81.66	0PS33ZZ+0PU33JZ ;0PS43ZZ+0PU43JZ; 0QS03ZZ+0QU03JZ; 0QS13ZZ+0QU13JZ; 0QSS3ZZ+0QUS3JZ		Percutaneous vertebral augmentation
Fusion				
Cervical fusion	81.01	0RG0xyz(y=7,J, K,Z,A; z=0,1,J) total=45	22548,22551,22554,22595,22590, 22600	Atlas-axis spinal fusion
	81.02	0RGsxy0(s=1,2,4; y=7,J,K,Z,A) total=45		Other cervical fusion of the anterior column, anterior technique
	81.03	0RGsxy1(s=1,2,4; y=7,J,K,Z,A) total=45		Other cervical fusion of the posterior column, posterior technique
		0RGsxyJ(s=1,2,4; y=7,J,K,Z,A) total=45-Posterior Approach, Anterior Column		Cervical fusion
Thoracic fusion	81.04	0RGsxy0(s=4,6,7,8,A; y=7,J,K,Z,A) total=75	22532, 22554, 22556,22610	Dorsal and dorsolumbar fusion of the anterior column, anterior technique
	81.05	0RGsxy1(s=4,6,7,8,A; y=7,J,K,Z,A) total=75		Dorsal and dorsolumbar fusion of the posterior column, posterior technique
		0RGsxyJ(s=4,6,7,8,A; y=7,J,K,Z,A) total=75		Thoracic fusion
Lumbar fusion	81.06	0SGsxy0(s=0,1,3; y=7,J,K,Z,A) total=45;-Fusion of Lumbar Vertebral Joint, Anterior Approach, Anterior Column, Open Approach	22533,22558,22586, 22612,22630,22633, 0195T	Lumbar and lumbosacral fusion of the anterior column, anterior technique
		0RGAxy0(y=7,J,K,Z,A) total=15; -Fusion of Thoracolumbar Vertebral Joint, Anterior Approach, Anterior Column, Open Approach		
	81.07	0SGsxy1(s=0,1,3; y=7,J,K,Z,A) total=45; -Fusion of Lumbar Vertebral Joint		Lumbar and lumbosacral fusion of the posterior column, posterior technique
		0RGAxy1(y=7,J,K,Z,A) total=15 -Fusion of Thoracolumbar Vertebral Joint,Posterior Approach, Posterior Column, Open Approach		
	81.08	0SGsxyJ(s=0,1,3; y=7,J,K,Z,A) total=45;-Fusion of Lumbar Vertebral Joint		Lumbar and lumbosacral fusion of the anterior column, posterior technique
		0RGAxyJ(y=7,J,K,Z,A) total=15; -Fusion of Thoracolumbar Vertebral Joint, Posterior Approach, Anterior Column		Lumbar fusion

**Table 8:** Summary of ICD-9/10 Primary Diagnosis Codes Utilized to Query Data from MarketScan, National Inpatient Sample and National Surgical Quality Improvement Program Databases International Classification of Diseases character 1 (i.e. E, J, N) refers to medical or surgical category designation and character 2 refers to body system, character 3 (i.e. 0B, 0G) refers to root operation.

Diagnosis	International Classification of Diseases-9 Code	International Classification of Diseases-10 Code	International Classification of Diseases-9 Description
Spinal stenosis	723.0	M48.01, M48.02, M48.03, M99.21-M99.71	Spinal stenosis in cervical region
	724.00	M48.00,	Spinal stenosis, unspecified region
	724.01	M48.04, M48.05, M99.22-M99.72	Spinal stenosis, thoracic region
	724.02	M48.06, M48.07, M99.23- M99.73	Spinal stenosis, lumbar region, without neurogenic claudication
	724.03 -claudication		Spinal stenosis, lumbar region, with neurogenic claudication
	724.09	M48.08, M99.24-M99.74	Spinal stenosis, other region
Disk herniation	722.0	M50.2x	Displacement of cervical intervertebral disc without myelopathy
	722.10	M51.26, M51.27	Displacement of lumbar intervertebral disc without myelopathy
	722.11	M51.24, M51.25	Displacement of thoracic intervertebral disc without myelopathy
Disc protrusion	722.30		Schmorl’s nodes, unspecified region
	722.31	M51.44, M51.45	Schmorl’s nodes, thoracic region
	722.32	M51.46, M51.47	Schmorl’s nodes, lumbar region
	722.4	M50.3x	Degeneration of cervical intervertebral disc
	722.51	M51.34, M51.35	Degeneration of thoracic or thoracolumbar intervertebral disc
	722.52	M51.36, M51.37	Degeneration of lumbar or lumbosacral intervertebral disc
	722.71	M50.0x	Intervertebral disc disorder with myelopathy, cervical region
	722.72	M51.04, M51.05	Intervertebral disc disorder with myelopathy, thoracic region
	722.73	M51.06, M51.07	Intervertebral disc disorder with myelopathy, lumbar region
Degenerative conditions	724.1	M54.6	Pain in thoracic spine
	724.3	M54.3x	Sciatica
	724.4	M54.14- M54.17	Thoracic or lumbosacral neuritis or radiculitis, unspecified
	724.5	M54.89, M54.9	Backache, unspecified
	724.9	M43.8x9, M53.80, M53.84, M53.85, M53.9	Other unspecified back disorders
	738.4	M43.0x, M43.1x	Acquired spondylolisthesis
	756.11	Q76.2	Spondylosis, lumbosacral region
	756.12	Q76.2	Spondylolisthesis
	756.19	Q76.41x, Q76.49	Other anomalies of spine
